# Physiological Chemistry

**Published:** 1905-12

**Authors:** John A. Miller


### PROGRESS IN MEDICAL SCIENCE.

### Physiological Chemistry.

By JOHN A. MILLER, Ph. D.

Chemical Changes in Bone Marrow after Intraperitoneal Injections of Bacteria, by Paul H. Müller, (Beit Chem. Physiol. Path. 1905; J. chem. soc., 88, 468). The composition of blood-plasma in normal rabbits is moderately constant; the average proteid-quotient (globulin : albumin) is 1 : 1.42. The injection of different virulent but dead cultures increases the fibrinogen and the total proteid of the blood-plasma; the serum globulin was not much affected. In the bone marrow, the total proteid and the fibrinogen were also much increased, especially by cultures of staphylococcus. The fibrinogen in this situation is so great that mere admixture with blood and lymph will not account for it all; its origin is believed to be the marrow.

Oxidation by the Urine, by K. Bertram, (Pflüger's Archiv., 1905; J. Chem. Soc., 88, 468). If a little indigo is added to urine, and then ferrous sulphate, the indigo is destroyed. If distilled water is used instead of urine, this does not occur. The oxidising action of the urine can also be demonstrated on Arsenious acid and other substances, and can be determined quantitatively. It is attributed to the presence of hydrogen peroxide.

Iron in Diabetic Urine, by S. Zucclu, (Zeit physiol. chem. 1905 J. C. Soc., 88, 469). Neumann and Mayer have described four cases of diabetes in which there was a constant relation between the sugar and the iron in the urine. This is important in view of the possible origin of sugar from nucleic acid. Three cases are, however, now recorded in which the proportion varied from 1.7 to 3.4 mg. iron per 100 grams of sugar.

Ether-glycosuria, and the Effect of Intravenous Oxygen Infusion on it, by A. Seelig, (chem. centr. 1905; J. C. Soc., 88, 469). Ether inhalations in dogs produce transient glycosuria, which is hindered by carbohydrate feeding. The glycosuria is accompanied by glycemia and a diminution of the hepatic glycogen. Simultaneous administration of oxygen intravenously, prevents the glycosuria.

Toxicity of Urinary Alkaloids, by H. Guillemard and P. Vranceano, (compt. rend. 1905; J. C. Soc., 88. 470). Under normal conditions, the alkaloidal toxicity varies between 18 and

25 per cent. of the "globale" toxicity of the urine. It is not proportional to the quantity of alkaloids, but depends on their nature. Creatinine is only slightly poisonous.

---

PHYSIOLOGICAL EFFECTS OF OZONE, by W. Sigmund, (Centr. Bakt. Par. 1905; J. C. Soc., 88, 476). Ozone retards the curdling of milk, but not sufficiently to be of use as a preservative. The employment of larger amounts of ozone would cause alterations in the composition of the milk. The rapid curdling of milk during thunder storms cannot be due to ozone, and is undoubtedly due in part to the higher temperature which prevails. It is suggested that the nitrous acid and oxides of nitrogen produced in storms may have something to do with it.

Mycoides and nodule-bacteria, when ozonised for an hour (with 1.3 mg. ozone) were not killed, but their developement was retarded.

Small amounts of ozone were found to be beneficial to the germination of peas, whilst larger amounts were injurious.

---

PURIFICATION AND STERILISATION OF DRINKING-WATER BY MEANS OF CALCIUM PEROXIDE, by L. Freyssinge and R. Roche, (Rev. intern. Falsif., 1905; J. C. Soc., 88, 575). The addition of about 0.4 gram of calcium peroxide per litre to water, completely sterilises the latter. The action is not instantaneous, but in most cases takes about two hours. After three hours no living bacteria were found in a water which originally contained 355,000 Bacilli coli-communis and 171,000 B. typhosus per c.c. The peroxide is more rapidly decomposed when a little sodium hydrogen carbonate is added to the water, and the sterilisation is then completed in about 15-20 minutes. The hydrogen peroxide resulting from the decomposition of the calcium peroxide may be removed together with the precipitate of calcium carbonate, by filtration through manganese dioxide. The hardness of the water is generally diminished by this treatment. If stored in closed bottles, the sterilised but unfiltered water can be kept for a long time before using for drinking.

---

ORIGIN OF LACTOSE. REMOVAL OF THE MAMMARY GLANDS DURING LACTATION, by C. Porcher, (Compt. rend. 1905., J. C. Soc., 88, 600). Soon after the operation, especially about the fourth and fifth hours, the urine contained much glucose (30-45 grams per litre). After 48 hours, however, (sometimes after 12-15 hours), the urine lost its reducing power.

Under normal conditions, the blood transports glucose to the mammary glands, where, in the regular course of lactation, it is converted into disaccharide and is excreted in the milk. The removal of the glands results in an accumulation of glucose in the blood, from which it passes to the urine. The rapid cessation of the transportation of glucose to the urine is attributed to a dimunition of the activity of the liver.

The glucose found in the urine is not accompanied by galactose.

---

So-called Normal Arsenic, by A. J. Kunkel, (Zeit. physiol. Chem. 1905; J. C. Soc., 88, 542). Gautier's statement that arsenic must be regarded as a normal constituent of animal tissues and organs is questioned. In the present research it was never found in any organs, even in the thyroid, to which Gautier attaches special importance.

